# Comprehensive exploration of the involvement of cuproptosis in tumorigenesis and progression of neuroblastoma

**DOI:** 10.1186/s12864-023-09699-2

**Published:** 2023-11-27

**Authors:** Rui Zhou, Dongmei Huang, Wen Fu, Fangpeng Shu

**Affiliations:** 1 Department of Urology, Guangzhou Women and Children’s Medical Center, National Children’s Medical Center for South Central Region, Guangzhou Medical University, Guangzhou, Guangdong China; 2 Department of Urology, Zhujiang Hospital, Souther Medical University, Guangzhou, Guangdong China; 3 Department of Thoracic Surgery, Nanfang Hospital, Souther Medical University, Guangzhou, Guangdong China; 4 Department of Pediatric Thoracic Surgery, Guangzhou Women and Children’s Medical Center, National Children’s Medical Center for South Central Region, Guangzhou Medical University, Guangzhou, Guangdong China

**Keywords:** Cuproptosis, Neuroblastoma (NB), PDHA1, Tumor immune microenvironment (TIME), Cell cycle

## Abstract

**Background:**

Copper-induced cell death, or “cuproptosis,” as an apoptotic process, has recently received much attention in human diseases. Recent studies on cuproptosis have provided novel insights into the pathogenesis of various diseases, especially cancers. However, the association between neuroblastoma (NB) and cuproptosis in terms of their clinical outcomes, tumorigenesis, and treatment response remains unclear.

**Methods:**

To determine the role of cuproptosis in NB tumorigenesis and progression, this study employed a systematic technique to explore the characteristic patterns of 10 key cuproptosis-related genes (CUGs) in NB. Consensus clustering analysis of the TARGET and GEO databases divided the NB patients into two subgroups that showed different clinicopathological attributes, molecular patterns, survival outcomes, disease-associated pathways, tumor immune microenvironment (TIME) features, and treatment responses. Moreover, a cuproptosis scoring scheme was established, which divided the patients with NB into two groups with high scores and low scores as per the median score. Furthermore, this research developed a nomogram and risk signature on the basis of this cuproptosis score to better elucidate its function in predicting NB prognosis. In vitro experiments were carried out using Transwell Assay, HLECs tube formation assay, Colony formation assay, Western Blotting Assay, Immunohistochemical (IHC) Staining, Immunofluorescence (IF) Staining and Flow Cytometry Analysis.

**Results:**

The results demonstrated that the established cuproptosis score and prediction model could effectively distinguish between the individuals in low and high-risk groups and had a high predictive value. Lastly, bioinformatics analysis and in vitro experiments enabled the identification of PDHA1, a key CUG, which was involved in both DNA replication-related pathways and the cell cycle. It was also associated with tumorigenesis and progression of NB.

**Conclusion:**

Cuproptosis, especially PDHA1, play a crucial role in the TIME characteristics, tumor progression, and long-term prognosis of NB. The patterns of cuproptosis assessed in this research may improve the understanding of the overall concept of NB tumorigenesis, thus facilitating the development of more effective therapeutic interventions.

**Supplementary Information:**

The online version contains supplementary material available at 10.1186/s12864-023-09699-2.

## Introduction

Neuroblastoma (NB) is the most prevalent type of extracranial cancer in children as well as the most prevalent tumor in infants and young children [1]. Even though the underlying etiology of this disease is still not fully understood, modifications in certain driver genes, such as ALK and MYCN, were recognized as significantly related to NB development. Owing to the extensive tumor heterogeneity, the prognosis of different NB patients is different [2, 3]. Moreover, NB may be classified into various groups on the basis of risk factors, i.e., low, intermediate, and high-risk groups [4]. The low-risk NB (most common in infants) has a good prognosis and can be managed by simple observation or surgical treatment; however, high-risk NB has a poor prognosis even with various intensive treatment regimens [3]. Therefore, early diagnosis and timely treatment of NB are particularly critical for managing the condition.

Copper-induced cell death, namely cuproptosis, which was suggested by Tsvetkov et al., is triggered by proteotoxic stress due to the accumulation of lipoylated dihydrolipoamide S-acetyltransferase (DLAT) as a direct result of excess intracellular copper [5]. Thus far, seven promoters, including PDHA1 (pyruvate dehydrogenase E1 component subunit alpha), FDX1, LIAS, LIPT1, DLD, PDHB, and DLAT, as well as 3 suppressors, including GLS, MTF1, and CDKN2A, are thought to be involved in the cuproptosis process. GLS has been identified as positively linked to both progression and tumorigenicity of NB, while the lack of CDKN2A expression is significantly correlated with poor prognosis and drug resistance in NB patients [6–8]. However, the mechanisms of other cuproptosis-related genes (CUGs) in NB are still unclear.

As a novel cell death modality, cuproptosis has recently been reported to play different roles in different types of tumors. Therefore, cancer risk models based on CUGs can be used for diagnostic and prognostic monitoring of several malignancies. For instance, hepatocellular carcinoma patients in the high cuprotosis-related risk score had a high mutational frequency of some tumor suppressors such as tumor protein P53 (TP53) and Breast-cancer susceptibility gene 1 (BRCA1)-associated protein 1 (BAP1) and a low frequency of catenin beta 1 (CTNNB1) [9]. Unsupervised cluster analyses based on CUGs may provide new strategies for the precision treatment of particular cancer patients. The high cuproptosis score group in bladder cancer may be more sensitive to chemotherapy, whereas the low cuproptosis score group is more sensitive to anti-PD1 immunotherapy [10]. In melanoma, high expression of LIPT1 might be an indicator of the favorable prognosis of melanoma after immunotherapy [11]. Furthermore, the function of non-coding RNAs associated with cuproptosis in the diagnosis as well as treatment of various cancers has also been extensively studied [12–14]; however, its role in NB remains uncertain. Therefore, exploring the role of cuproptosis in the pathogenesis and treatment of NB is crucial for fully understanding the concept.

This study aimed to establish several patterns of cuproptosis associated with distinct tumor immune microenvironment (TIME) and prognostic features. Furthermore, the use of cuproptosis scores was proposed to quantify these patterns in each NB patient. This scoring system proved helpful in establishing a more accurate, robust, and personalized approach to the treatment of NB. Additionally, the oncogenic role of PDHA1 in NB was also highlighted via both in vitro experiments and bioinformatics analysis, which facilitated further insight into the underlying mechanism.

## Materials and methods

### Data collection

Normalized data for gene expression (in Fragments/Kilobase of sequence/Million mapped reads [FPKM] format) and the comparable overall survival (OS) and clinicopathological data of the patients were collected from the GSE49710 dataset in GEO (n = 498, https://www.ncbi.nlm.nih.gov/geo/) and the GDC TARGET-NB datasets in UCSC Xena (n = 150, https://xena.ucsc.edu/). Of these, patients who missed any important survival or clinical parameters were not included in further analysis. The R package “limma 3.52.2” was applied to normalize the raw reads of the data.

### Consensus clustering analysis

A summary of the basic information of the 10 CUGs was acquired from https://www.ncbi.nlm.nih.gov/gene and were listed in Supplementary table S1. The expression profiles of the 10 CUGs were used as a reference, and the “ConsensusClusterPlus” package in R was used for consensus unsupervised clustering analysis. The individuals with NB from the TARGET database were subsequently clustered into specific molecular subgroups. The *k*-means algorithm was applied to calculate the optimal grouping number, and finally, t-Distributed Stochastic Neighbor Embedding (t-SNE) was employed for verifying the clusters as per the available expression profiles of the aforementioned selected genes.

### Association of cuproptosis patterns with clinicopathological features and prognosis of NB individuals

To explore the clinical significance of different cuproptosis patterns, a comparison of the associations was done between clinicopathological attributes, cuproptosis patterns, and survival outcomes in NB. The Kaplan–Meier (KM) curve was applied to determine the OS between distinct NB groups. Also, the gene set variation analysis (GSVA) was conducted via the “GSVA” package in R and it helped identify the variations in the biological roles across different cuproptosis patterns.

### Characterization of differentially expressed genes (DEGs)

The “limma” package in R was employed for recognizing DEGs in the different molecular subgroups from the CUG clustering. Genes with an adjusted *p*-value < 0.05 and |log2 fold change (FC)| >0.585 were assumed to have significant differential expression. Of these DEGs, the genes involved in prognosis were further analyzed.

### Cuproptosis score calculation

The calculation of cuproptosis scores enabled the quantification of the cuproptosis patterns of each NB patient. First of all, the Boruta algorithm was applied to reduce the size of the cuproprosis gene signatures A and B. Also, principal component 1 was extracted via principal component analysis (PCA) as the signature score. Thereafter, patient scores were calculated using the following equation: cuproptosis score = *∑*PC1A − *∑*PC1B, where PC1A depicts the first component of feature A and PC1B depicts the first component of feature B. Moreover, the Kruskal–Wallis test was then applied to compute the cuproptosis scores for gene clusters or molecular patterns.

### Association between cuproptosis score, clinical significance, and drug sensitivity

The Chi-square test was employed to assess the association between the cuproptosis score and clinical features of NB like gender, age, state of survival, risk, and stage. Furthermore, the predictive capacity of the cuproptosis score was stratified in different clinical characteristics. Univariate and multivariate Cox proportional hazards regression (CPHR) analyses were also conducted to ascertain if the cuproptosis score values could serve as independent predictors of NB prognosis. Additionally, the calibration plots and nomogram were generated to determine the clinical accuracy of the score. Furthermore, the GSEA enrichment analysis (at |log2 FC| ≥1 and *p*-value < 0.05) was conducted for both groups to determine candidate cuproptosis-related pathways in NB.

### Human tissue sample

A total of 20 paraffin-embedded, formalin-fixed NB samples were obtained from the patients undergoing surgery at the Guangzhou Women and Children’s Medical Center (Guangzhou, China) between April 2016 and August 2022, ensuring explicit written consent. All the samples were pathologically confirmed as NB by two independent pathologists. Ethical approval was obtained from Guangzhou Women and Children’s Medical Center’s Committee for the Ethical Review of Research Involving Human Subjects.

### Cell culture

The NB cell lines, SH-SY5Y and SK-N-AS, as well as the human lymphatic endothelial cells (HLECs), were retrieved from the type culture collection of the Chinese Academy of Sciences (Shanghai, China). SK-N-AS cells were cultured in Dulbecco’s Modified Eagle Medium (DMEM) supplemented with 1% penicillin-streptomycin and 10% fetal bovine serum (FBS, Gimini, Calabasas, CA, USA). The SH-SY5Y cells, on the other hand, were cultured in Minimum Essential Medium Eagle/Nutrient Mixture F12 Ham (MEM/F12, PM151220, Procell, Wuhan, China) supplemented with 1% penicillin-streptomycin and 15% FBS (Gimini, Calabasas, CA, USA). HLECs were cultured in Endothelial Cell Medium (ECM, Procell, Wuhan, China) supplemented with 10% FBS (Gimini, Calabasas, CA, USA) and 1% penicillin-streptomycin. Following this, all cells were incubated at 5% CO_2_ and 37 ℃ in a humidified environment.

### Cell transfection

SH-SY5Y and SK-N-AS cells were transfected with the aid of Lipofectamine 3000 (Invitrogen; Thermo Fisher Scientific, Inc, USA) as per the manufacturer’s instructions. Small interfering RNAs (siRNA) targeting PDHA1, MTF1, GLS, PDHB and negative control (Supplementary Table S2) were supplied by KeScience (Shanghai, China).

### Quantitative real-time polymerase chain reaction (qRT-PCR)

Total RNA was extracted from tissues and then reverse transcripted to produce complementary DNA (cDNA) using a high-capacity cDNA reverse transcription kit (Applied Biosystems, California). QRT-PCR methods referred to previous published paper [15]. The following primers were used: PDHA1, forward 5′-GGACGCCGTTCTGGTTG-3′ and reverse 5′-CTTTCCGTGTCCTGTAACCC-3′; MTF1, forward 5′-GGCAAAGCCTTCCTTACCTCT-3′ and reverse 5′-ACTGAGTGTGGTGAAGTATTTGCTG-3′; GLS, forward 5′-TTCCAGAAGGCACAGACATGGTTG′ and reverse 5′-GCCAGTGTCGCAGCCATCAC-3′; PDHB, forward 5′-AAGAGGCGCTTTCACTGGAC-3′ and reverse 5′-ACTAACCTTGTATGCCCCATCA-3′; β-actin, forward 5′-CCCGAGCCGTGTTTCC-3′ and reverse 5′-GTCCCAGTTGGTGACGATGC-3′. All primers were purchased from RiboBio Co. Ltd. (Guangzhou, China). The relative RNA abundance was determined using the 2^−ΔΔCt^ method.

### Transwell Assay

Full culture medium (500 µl) was added to the bottom wells, whereas the transfected cells (2 × 10^5^) with serum-free DMEM were placed in the upper wells (8 μm membrane). Following a 24-hour incubation at 37 ℃, the cells were removed from the upper surface, and the lower surface wells were fixed using methanol. Thereafter, the cells were stained using crystal violet (0.1%) dye and photographed under a 100× microscope (Olympus, Japan).

### HLECs tube formation assay

The 24-well plates were prepared by the addition of 200 µl extracellular matrix (ECM) mixed with 100 µl Matrigel, followed by incubation for 12 h. Next, the 50 µl ECM containing HLECs (5 × 10^5^) were seeded into the plates and incubated for another hour, after which the 250 µl serum-free media was collected from the tumor cell culture and added to the wells. After another 4 h of incubation, the resultant lymphatic tubes were photographed with the aid of an inverted microscope.

### CCK-8 assay and colony formation assay

Cells were seeded into 96-well plates (4000 cells/well) and cultured for 24 h. After cells were supplemented using Cell Counting Kit-8 (CCK-8) (Dojindo, Kumamoto, Japan) for 60 min, wavelength of 450 nm was selected to detect the absorbency of cells.

Transfected cells were seeded into 6-well plates (500 cells/well) and then incubated in DMEM supplemented with 10% FBS for 7–14 days. The colonies were fixed using 4% paraformaldehyde, stained with crystal violet (0.1%) dye, and finally counted.

### Western blotting assay

The assay was performed as described earlier [15]. The primary antibodies used in this study included anti-PDHA1 (bs-4034-R, Bioss) and anti-β-actin (ab6276, Abcam).

### Immunohistochemical (IHC) staining

The expression profiles of PDHA1 in NB tissues were quantified using anti-PDHA1 (bs-4034-R, Bioss) antibodies. The assay was performed as described earlier [15].

### Immunofluorescence (IF) staining

NB tissue slides were incubated with anti-PDHA1, anti-neuron specific enolase (NSE) (AF5473, Affinity), and anti-CD56 (AF0931, Affinity) antibodies for an hour. Thereafter, the slides were again incubated with fluorescence-labeled secondary antibodies (Servicebio) and then observed using a fluorescence microscope (ECLIPSE TISR, Nikon). Moreover, the entire procedure was conducted at room temperature.

### Flow Cytometry Analysis for Cell Cycle

The transfected cells were stained by Cell Cycle and Apoptosis Analysis Kit (Beyotime Biotechnology, Shanghai, China) and subsequently detected using flow cytometry. Following this, the cell cycle was evaluated using a FACS Calibur flow cytometer (BD Biosciences, San Jose, CA, United States).

### Statistical analysis

Statistical analyses of the bioinformatics data were conducted using R 4.1.0. For the experimental data, statistical analyses were performed using GraphPad Prism (GraphPad, La Jolla, CA, USA). Statistical significance was set at a two-sided *p*-value of < 0.05. Differences between two groups were evaluated via unpaired or paired Student’s t-test (two-tailed tests), while differences across multiple groups were evaluated via the one-way ANOVA followed by Dunnett’s multiple comparison test. In addition, the chi-square (χ^2^) test was applied for non-parametric variables.

## Results

### Survival analysis of OS patients as per CUGs

**Fig. 1:**
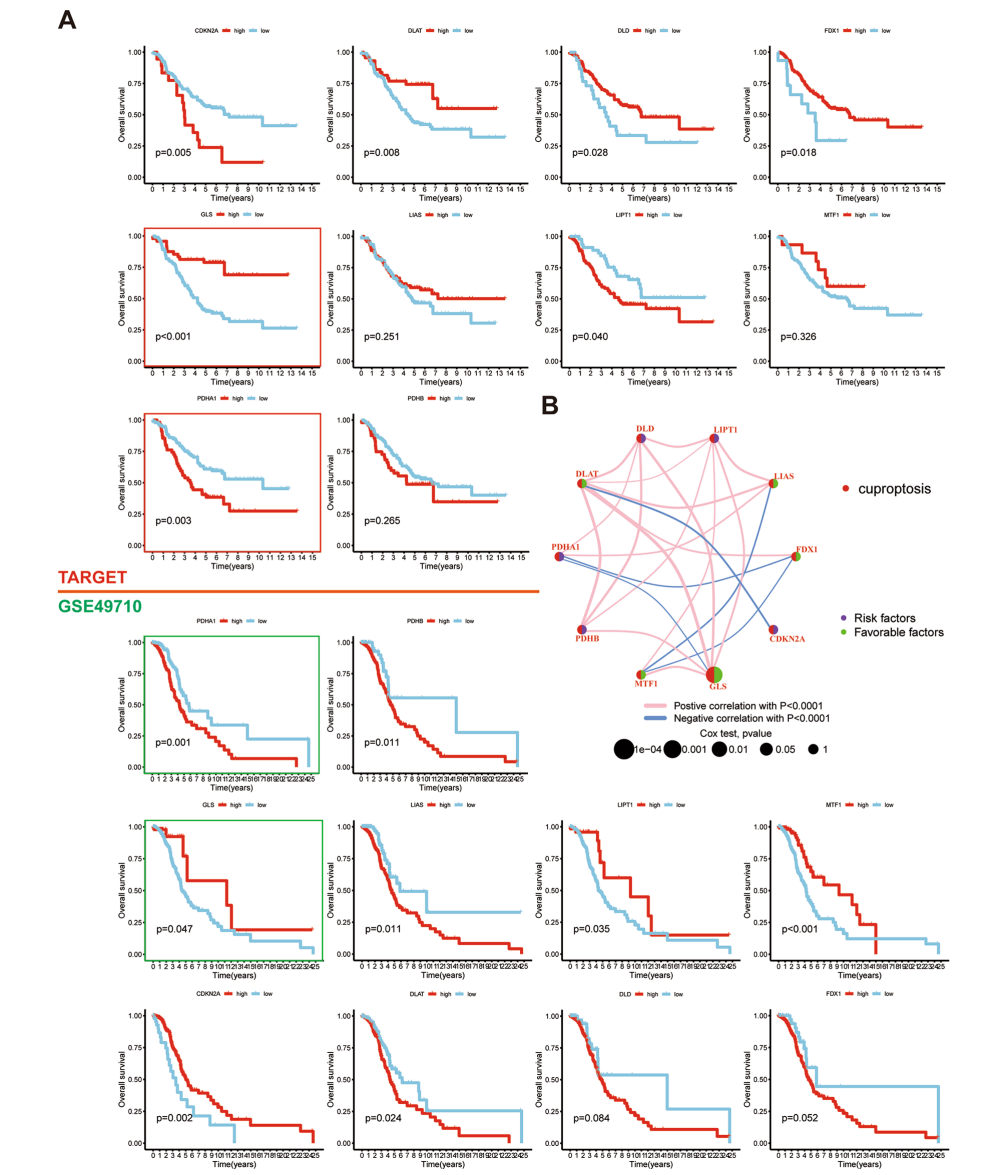
Effect of cuproptosis-related genes (CUGs) on neuroblastoma (NB) prognosis. (**A**) survival analysis of NB patients based on CUGs and (**B**) the regulatory relationships, interaction networks, and survival significance of CUGs in individuals with NB

To explore the relationship between NB prognosis and CUG, the study conducted survival analysis for each CUG in NB patients from the GSE49710 (n = 498) and TARGET (n = 150) datasets. Both PDHA1 and GLS showed consistent significant association with survival in the two datasets, while the other CUGs demonstrated either insignificant or inconsistent impact on NB survival (Fig. 1A). Thereafter, the study revealed regulator relationships, interaction networks, and survival significance of CUGs in individuals with NB (Fig. 1B), which were consistent with the survival analysis results.

### Identification of cuproptosis subtypes and their corresponding pathway and clinical features in NB

**Fig. 2:**
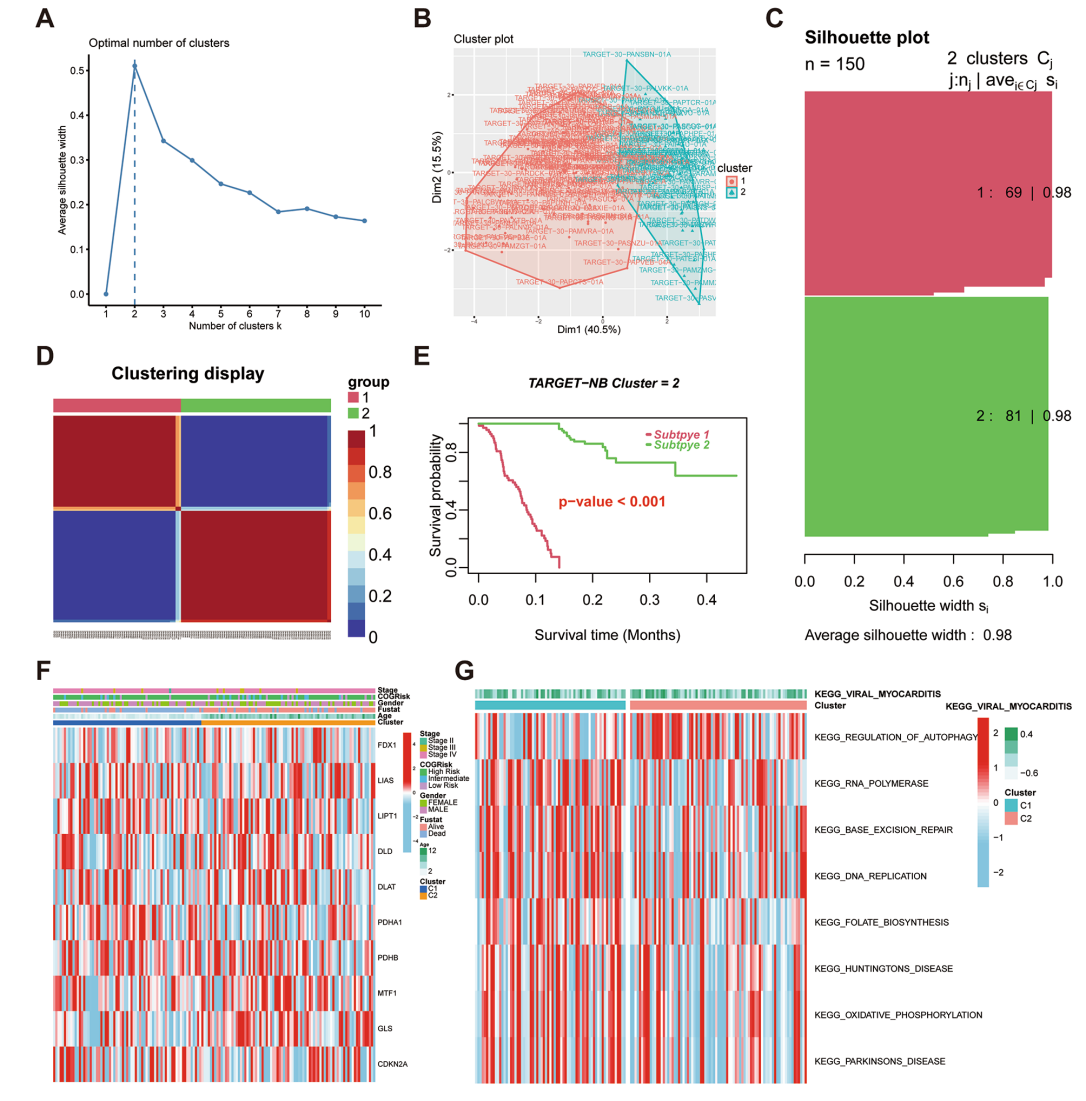
Cuproptosis subtypes and their corresponding clinical and pathway characteristics in NB. (**A**) unsupervised clustering of TARGET-NB samples according to the mRNA expression levels of the 10 cuproptosis-related genes (CUGs) (*k* index = 2); (**B**) t-Distributed Stochastic Neighbor Embedding (t-SNE) of the expression profiles of CUGs mRNAs in the TARGET-NB dataset stratified the samples into 2 groups: C1 and C2; (**C**) silhouette plot of the 2 groups; (**D**) clustering representation of the molecular subgroups; (**E**) Kaplan–Meier test curves of the molecular patterns of individuals with NB; (**F**) heatmap showing the association between the subtypes as well as different clinicopathological attributes; and (**G**) GSVA enrichment analysis test of biological pathways between C1 and C2

The study employed unsupervised non-negative matrix factorization (NMF) clustering on the TARGET-NB samples as per the mRNA expression levels of each of the 10 CUGs. Genetic correlation analysis and molecular clustering effect were applied to comprehensively ascertain the optimal *K*. Of all *k* values, *k* = 2 demonstrated the optimal clustering effect (Fig. 2A–C), and the two subgroups obtained by clustering were named Clusters 1 and 2 (C1 and C2, respectively) (Fig. 2D). KM survival analysis showed that the expectation of survival in the C1 group was considerably lower than in the C2 group (Fig. 2E). Moreover, the C2 group demonstrated both longer survival duration and better survival status than the other group. As shown in Fig. 2F and Figure S1, it was concluded that the correlation between the gene expression of CUGs and the clinical features of the clusters was significantly different. Moreover, GLS and PDHA1 were overexpressed significantly in the C2 and C1 groups, respectively. These results are consistent with the above results, thereby demonstrating the accuracy and overall effectiveness of the clustering scheme. Additionally, the GSVA results showed that the C1 group was enriched in tumorigenesis-associated pathways, like DNA-replication and RNA-polymerase signaling pathways, whereas C2 was significantly enriched in pathways associated with cell death, like the autophagy signaling pathway (Fig. 2G).

### Clinical and TIME characteristics of the cuproptosis gene clusters of NB

**Fig. 3:**
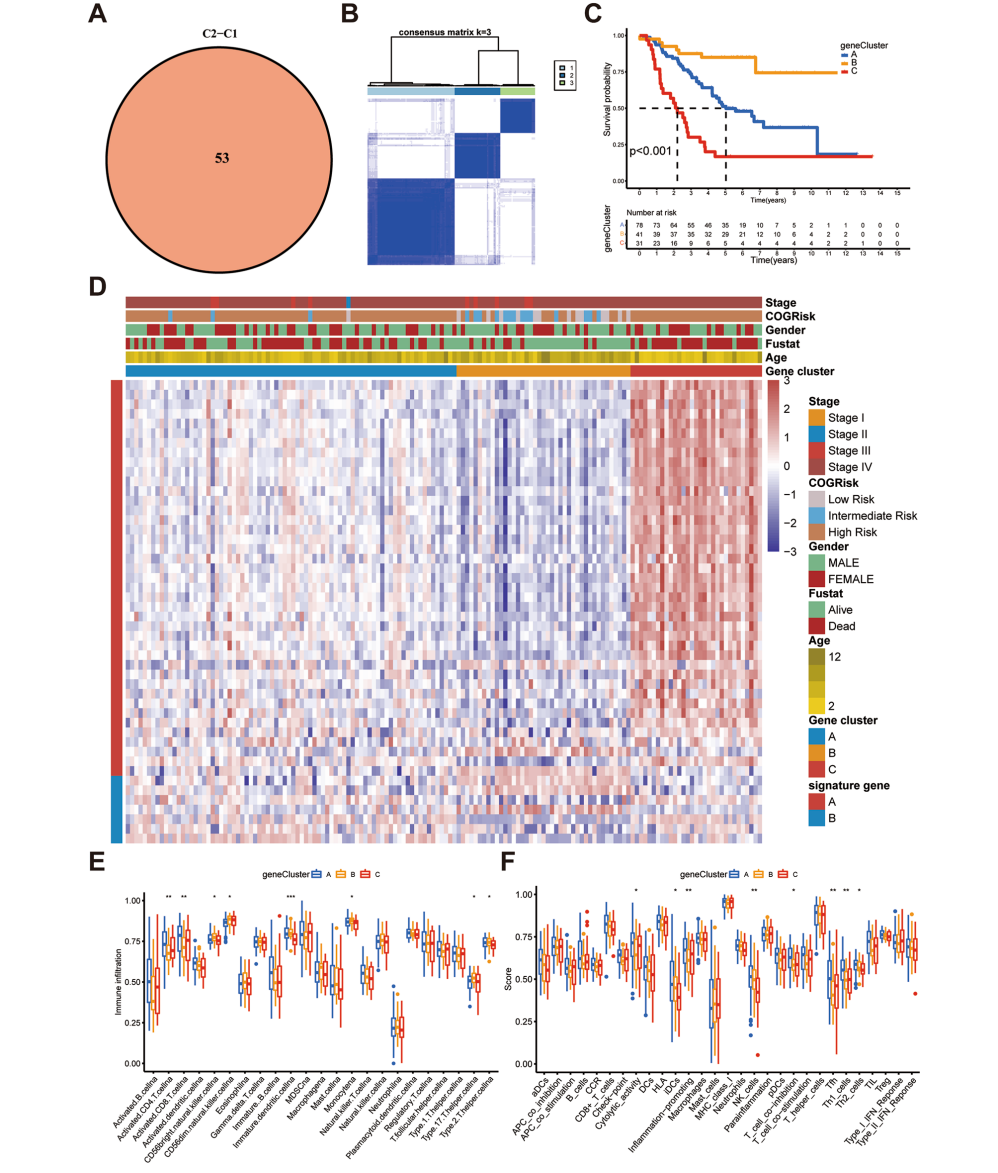
Different clinical and tumor immune microenvironment (TIME) features of the C1 and C2 subtypes in NB. (**A**) Venn diagram of the prognostic differentially expressed genes (DEGs) through pairwise comparison between the C1 and C2 subgroups; (**B**) consensus matrix heatmap depicting three gene clusters as per the prognostic DEGs; (**C**) Kaplan–Meier survival test of the NB patients in the gene groups A, B, and C; (**D**) clinical characteristics of the 3 cuproptosis gene clusters; and (**E, F**) boxplots showing (**E**) the abundance of 23 infiltrating immune cell types and (**F**) differences in immune scores in the 3 gene clusters

The study identified 53 prognostic DEGs by differential expression analysis of the two clusters with the aid of univariate CPHR and the “limma” package (Fig. 3A). Thereafter, the Target-NB cohort was classified into three sub-cohorts: gene clusters A, B, and C, as per the associated prognostic DEGs (Fig. 3B, Figure S2). The survival analysis results showed that the three gene clusters were significantly different in terms of their survival expectations (Fig. 3C). Additionally, the heatmap indicated that gene cluster 3 had poor survival and was more prevalent in younger patients (Fig. 3D). TIME analysis showed that gene cluster A had stronger immune function as well as a higher proportion of immune cell infiltration (ICI) (Fig. 3E, F). CD8 + and CD4 + T cells showed significant immune exhaustion in clusters B and C, which may be a crucial process of NB immune escape [16]. Hence, as per the consistency of TIME and prognostic characteristics of the three clusters, this classification method can be assumed credible and reasonable.

### Development and validation of cuproptosis scores for NB

**Fig. 4:**
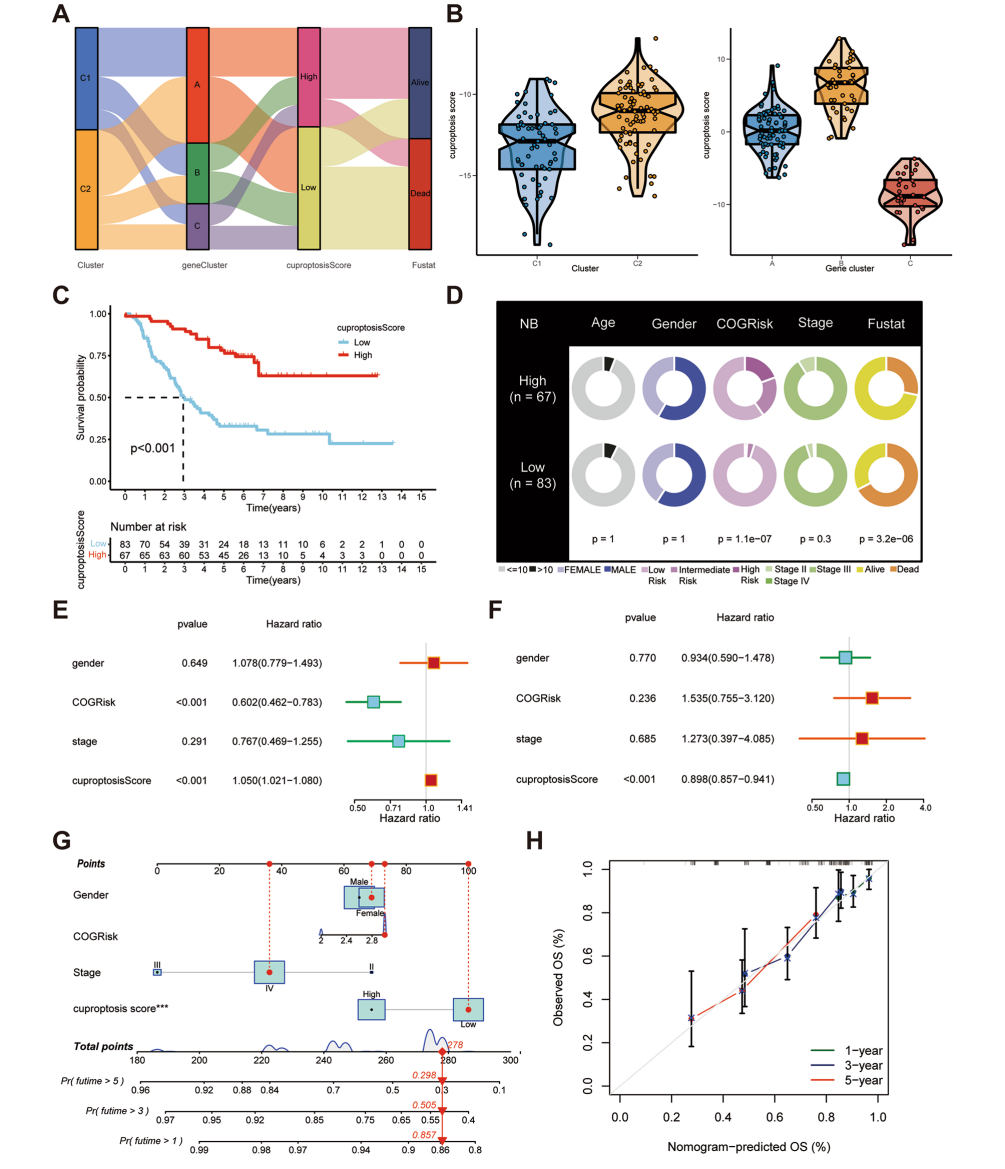
Development and validation of cuproptosis scores for neuroblastoma (NB). (**A**) alluvial diagram of 2 cuproptosis subtypes (high and low score), cuproptosis score values, 3 gene clusters, and clinical results; (**B**) differences in cuproptosis score values of the high and low-score subtypes as well as the gene clusters A, B, and C; (**C**) Kaplan–Meier analysis of the overall survival (OS) between the 2 cuproptosis score groups; (**D**) clinical features of the 2 cuproptosis score groups; (**E, F**) the (**E**) uniCox and (**F**) multiCox analyses demonstrating the independent prognostic value of the cuproptosis score and multiple clinical factors; (**G**) a nomogram for estimating the one, three, and five-year OS of individuals with NB in the TARGET-NB cohort; and (**H**) calibration curves for verifying the developed nomogram

The cuproptosis scores were calculated for each TARGET-NB sample based on the 53 prognostic DEGs through PCA and stratified the NB patients into high and low-score groups as per the median value. Figure 4 A shows the survival status of the patients as well as the differential distribution of cuproptosis scores across the two subgroups and three gene clusters. As shown in Fig. 4B, compared with the high-score group, the low-score group was associated with the subgroup or gene clusters that had a poor prognosis. These results were further confirmed by the subsequent survival analysis (Fig. 4C). Thereafter, the clinical factors of the NB patients in different groups were analyzed (Fig. 4D; Table 1). UniCox and multiCox analyses were performed to combine the cuproptosis scores with clinical factors (Fig. 4E, F), and after excluding the synergistic effects of other variables, the study concluded that the cuproptosis score could potentially serve as an independent prognostic factor for NB. A nomogram was subsequently constructed based on both clinical factors and cuproptosis score to predict the overall survival of NB patients (Fig. 4G), and its accuracy was cross-verified via calibration curves (Fig. 4H).

**Table 1:** Relationship between cuproptosis score and clinicopathological features of TARGET-neuroblastoma (NBL) dataset

Covariates	Total	High-score	Low-score	*P*-value
		No. (%)	No. (%)	
**Age (years)**				1
**≤** 10	140 (93.33%)	63 (94.03%)	77 (92.77%)	
> 10	10 (6.67%)	4 (5.97%)	6 (7.23%)	
**Gender**				1
Female	62 (41.33%)	28 (41.79%)	34 (40.96%)	
Male	88 (58.67%)	39 (58.21%)	49 (59.04%)	
**Status**				0.0005
Alive	75 (50%)	48 (71.64%)	56 (67.47%)	
Dead	75 (50%)	19 (28.36%)	34 (40.96%)	
**COGRisk**				0.0005
High Risk	119 (79.33%)	40 (59.7%)	79 (95.18%)	
Intermediate Risk	17 (11.33%)	14 (20.9%)	3 (3.61%)	
Low Risk	14 (9.33%)	13 (19.4%)	1 (1.2%)	
**TNM stage**				0.3058
II	1 (0.67%)	0 (0%)	1 (1.2%)	
III	9 (6%)	6 (8.96%)	3 (3.61%)	
IV	140 (93.33%)	61 (91.04%)	79 (95.18%)	

### The mechanisms of cuproptosis in NB pathogenesis and NB TIME

**Fig. 5:**
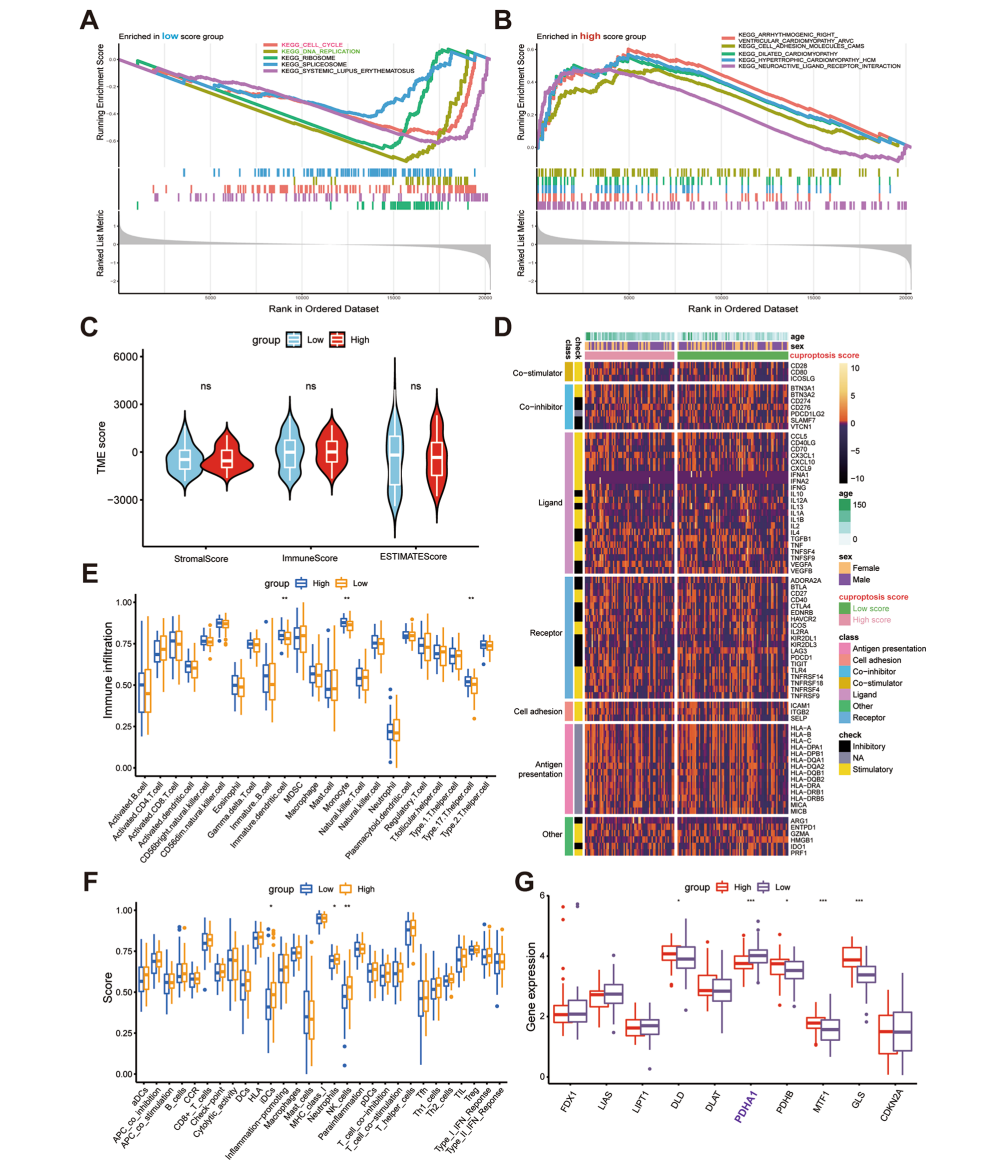
Distinct pathways and tumor immune microenvironment (TIME) characteristics of NB patients as per the cuproptosis score values. (**A, B**) GSEA displaying the significantly enriched pathways in the (**A**) low-score (**B**) high-score groups; (**C**) associations between the cuproptosis score, stromal scores, and immune scores; (**D**) thermogram depicting varying expression of various cytokines among the 2 cuproptosis score groups; (**E, F**) boxplots showing (**E**) the abundance of 23 infiltrating immune cell types and (**F**) differences in the immune scores among the two cuproptosis score groups; and (**G**) differences in the expression of 10 cuproptosis-linked genes between the two cuproptosis score groups. (p < 0.05 *; p < 0.01 **; p < 0.001 ***)

To determine the molecular mechanism of cuproptosis in NB-related pathogenesis, the pathways enriched in both the high and low-score groups were identified, and the top five pathways were screened using the normalized enrichment score (NES) values. As shown in Fig. 5A and B, the low-score group was more significantly enriched in tumorigenesis-associated pathways, like cell cycle and DNA replication signaling pathways, compared to the high-score group. The TIME can modulate the progression of various cancers [17], and NB exists in a complex TIME. ICI and various inflammatory factors play key roles in tumor development and significantly affect NB prognosis [18]. However, the study did not find any significant differences in the distribution of immune or stromal cells between both groups (Fig. 5C). Moreover, the two groups also did not exhibit significant differences in the inflammatory cytokine expression levels (Fig. 5D). Further analysis of immune function and ICI differences also supported that TIME was weakly associated with the curpoptosis score (Fig. 5E, F), pointing that cuproptosis was primarily involved in tumor cell proliferation-associated pathways in NB, as evidenced by GSEA enrichment analysis, but had no significant influence on TIME. To detect the key CUGs involved in NB tumor cell proliferation, the study analyzed the differential expression of 10 major CUGs between the high and low-score groups (Fig. 5G) and concluded that only PDHA1 was significantly upregulated in the low-score group. Thus, it was hypothesized that PDHA1 could be a potential key CUG in NB tumorigenesis. The mRNA level of PDHA1 in NB tissues was found positively correlated with the tumor stages through qRT-PCR (Figure S3**A**).

### General characteristics of PDHA1 in pan-cancer and NB

**Fig. 6:**
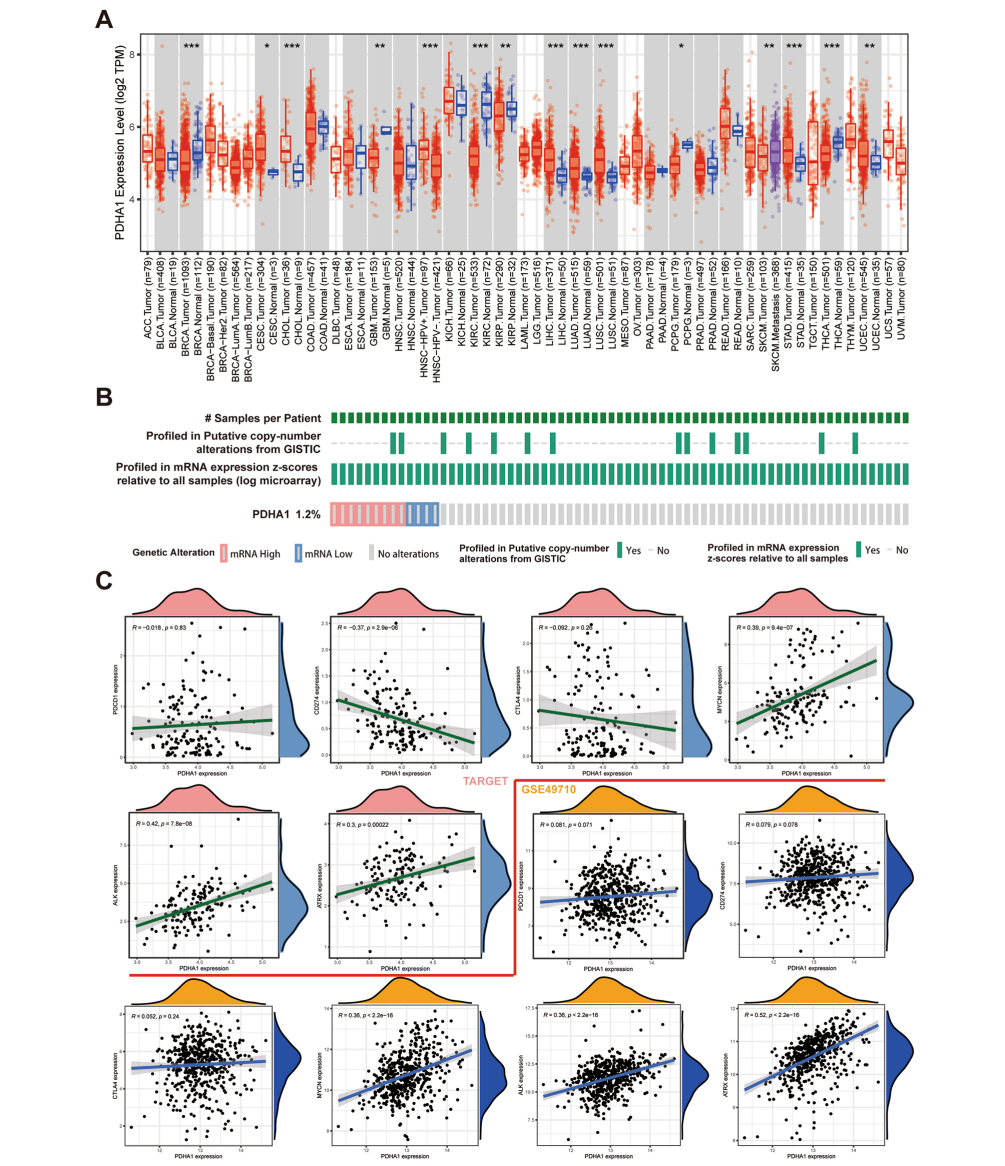
Exploration of the general characteristics of PDHA1 in NB and pan-cancer. (**A**) differential mRNA expression of PDHA1 in pan-cancer; (**B**) mutation landscape of PDHA1 in NB; and (**C**) the interaction between PDHA1 and immune checkpoints and some key genes (MYCN, ALK, and ATRX) in NB. (p < 0.05 *; p < 0.01 **; p < 0.001 ***)

Based on previous findings of the key role of PDHA1 in NB pathogenesis, this study further explored its role in pan-cancer. Differential expression in pan-cancer was analyzed by using the Timer 2.0 database (http://timer.cistrome.org/) and concluded that it differed remarkably across different types of tumors (Fig. 6A), indicating that PDHA1 is multifaceted. Also, the mutation landscape in the cBioPortal database (https://www.cbioportal.org/) displayed aberrant mRNA expression of PDHA1 in NB (Fig. 6B). Additionally, dysregulation of the MYCN transcription factor is frequently found in NB. MYCN oncogene amplification is detected in 25% of NB patients and is a known marker of tumor aggressiveness [19]. ALK is the most common somatic mutated gene in NB found in around 14% of the high-risk tumors and around 9% of the primary NB tumors [20, 21]. Furthermore, ALK amplification has been reported to be almost exclusively associated with MYCN co-amplification [22, 23]. ATRX alterations have also been reported to occur at a high frequency in adolescents and young adults with NB [24]. Therefore, several studies have been conducted on these genes as targets for NB treatment [25–27]. Therefore, it is crucial to explore the interaction of these key genes with PDHA1 for the treatment of NB. Co-expression analysis based on TARGET-NB and GSE49710 datasets revealed that PDHA1 was correlated positively with the mRNA expression levels of MYCN, ALK, and ATRX in NB (Fig. 6C), indicating the potential oncogenic role of PDHA1 in NB. Moreover, correlation analysis with PD1 (PDCD1), PDL1 (CD274), and CTLA4 further proved that PDHA1 had no significant correlation with NB TIME (Fig. 6C).

### PDHA1 is involved in cell cycle and proliferation-related pathways in NB

**Fig. 7:**
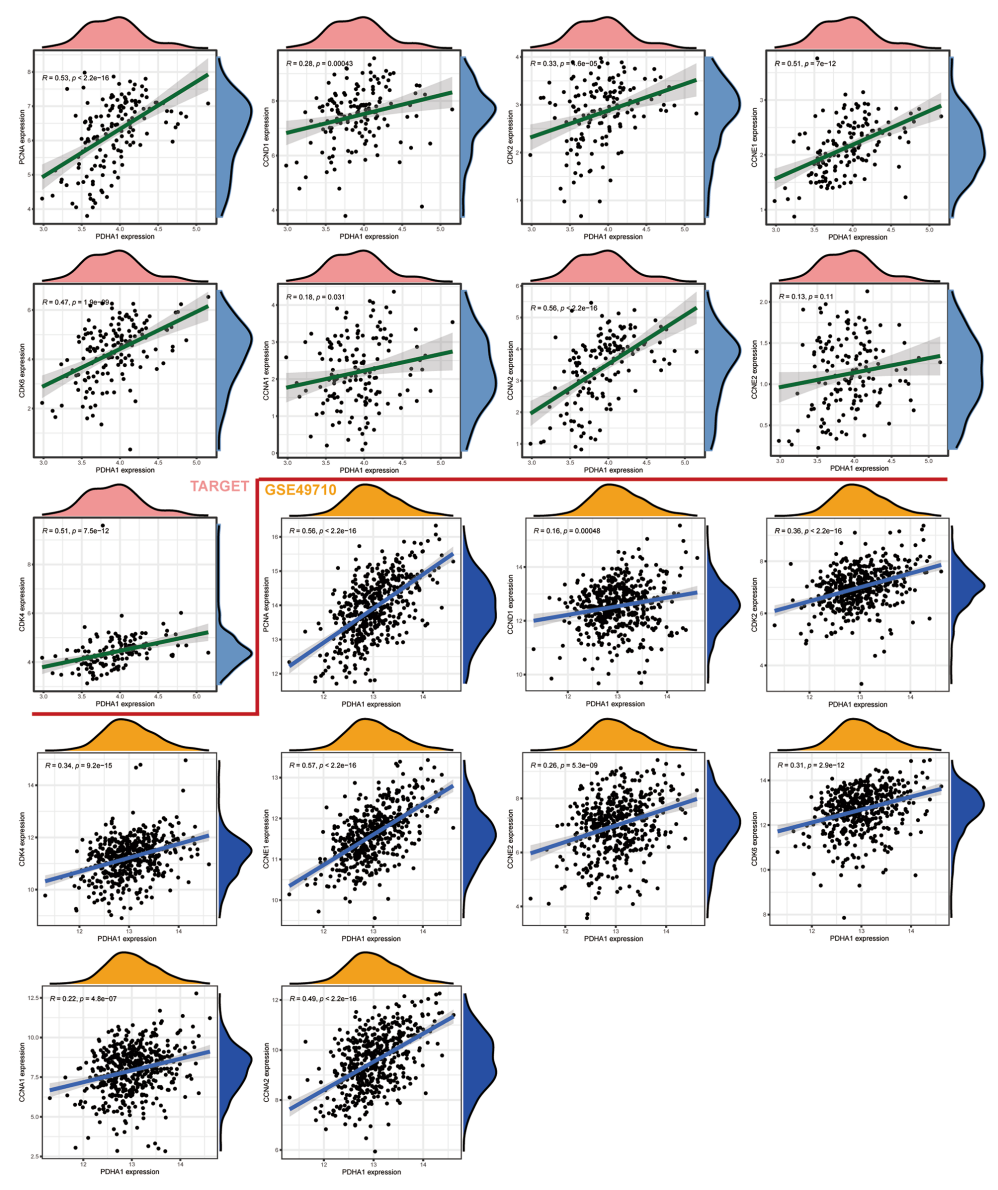
PDHA1 was positively correlated with checkpoints of the cell cycle and proliferation-related pathways

Based on the above findings, it was hypothesized that PDHA1 might be involved in tumorigenesis and progression of NB via the cell cycle and proliferation-related pathways. Therefore, the relationship between PDHA1 and the cell cycle and proliferation-associated markers, including PCNA, CCND1, CDK2, CDK4, CDK6, CCNA1, CCNA2, CCNE1, and CCNE2, was investigated. Co-expression analysis revealed that PDHA1 was correlated positively with these cancer-associated pathway markers (Fig. 7). The evidence from bioinformatic analyses indicated that PDHA1 might influence NB onset and progression by participating in proliferation-associated pathways and the cell cycle.

### PDHA1 is correlated with tumor stages and natural killer (NK) cell infiltration in NB

**Fig. 8:**
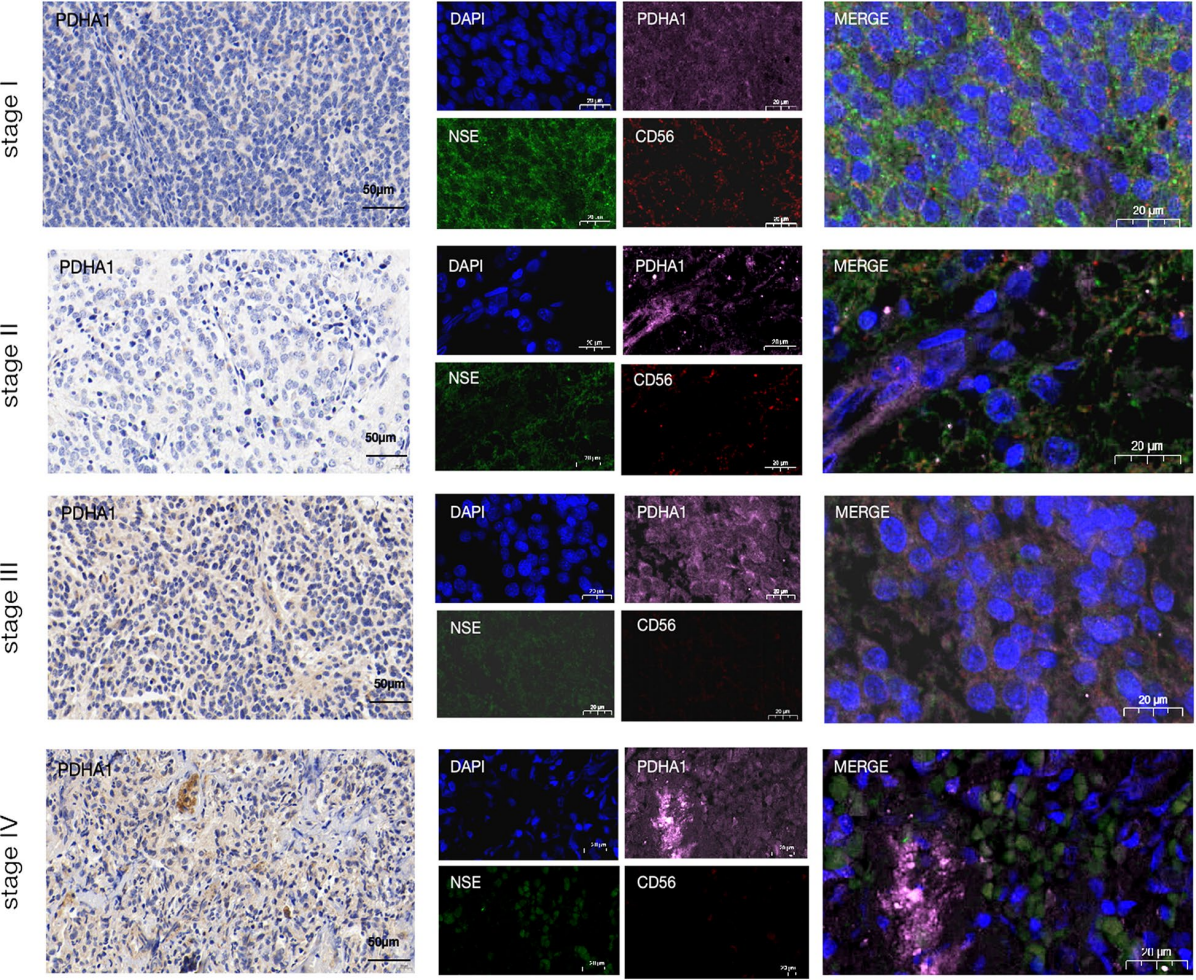
PDHA1 was correlated with tumor stages and natural killer (NK) cell infiltration of neuroblastoma (NB)

As seen in Fig. 5E, NK cell infiltration level was of no significant difference in the two groups. But according to Fig. 5F, the NK cells exerted more important functions in high-score groups. To explore the potential relationship between PDHA1 and NK cell infiltration, IHC and multiple IF assays were performed in different tumor stages. The results (Fig. 8) revealed that tumor tissue in higher stages expressed higher PDHA1 and lower CD56, indicating increased tumor proliferation and decreased NK cell infiltration.

### PDHA1 promotes proliferation, invasiveness, and lymphatic metastasis of NB cells in vitro via the cell cycle pathway

**Fig. 9:**
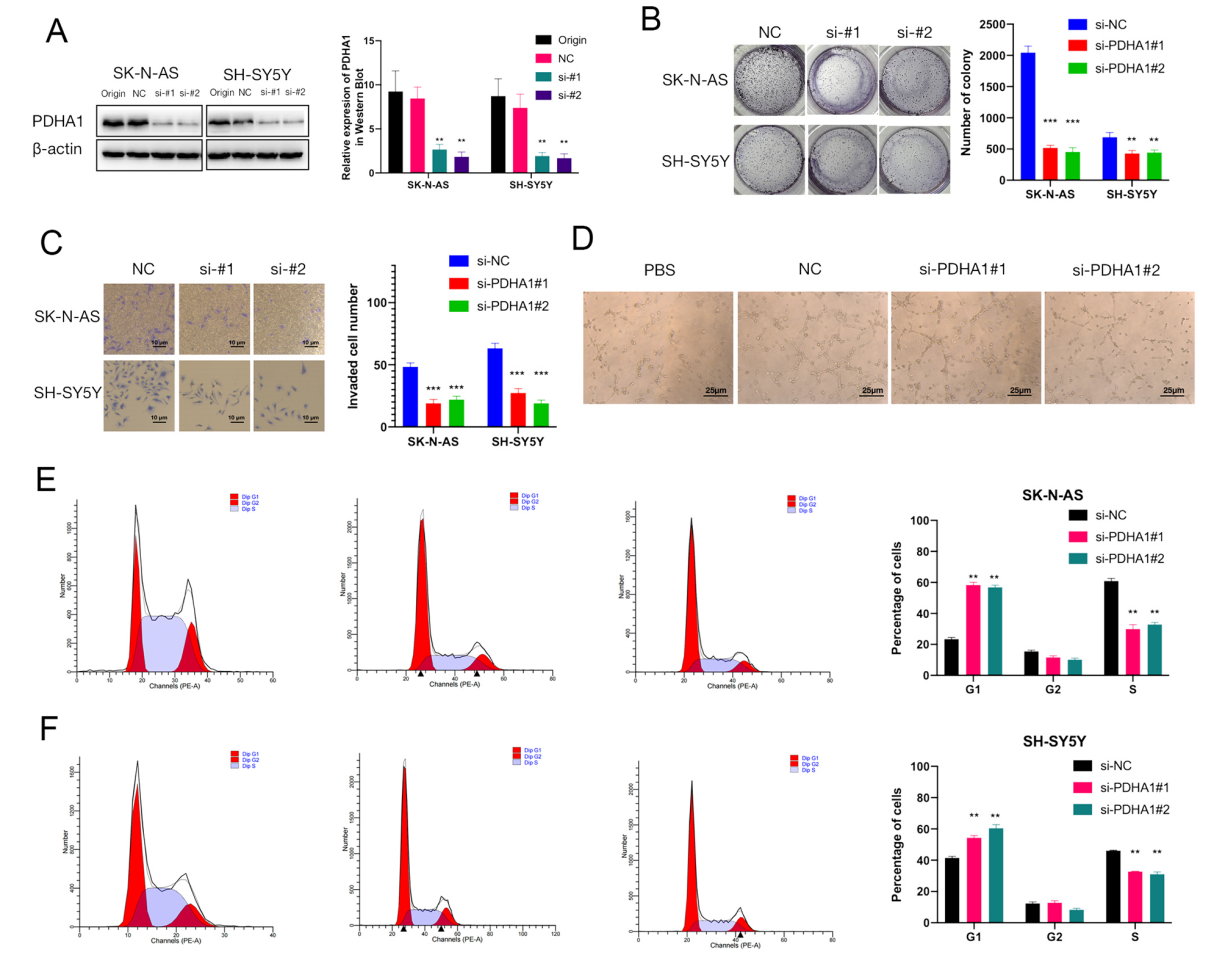
PDHA1 promotes proliferation, invasiveness, and lymphatic metastasis of neuroblastoma (NB) cells in vitro via cell cycle pathway (**A**) western blot analysis of NB cell lines after transfection with si-PDHA1#2 and si-PDHA1#1 to decrease the expression level of PDHA1; (**B**) cell colony formation efficiency of the knockdown of PDHA1 NB cells as well as control cells; (**C**) invasiveness of PDHA1 knockdown NB cells and control cells; (**D**) lymphoangiogenetic efficiency of HLECs incubated with the culture medium from PDHA1 knockdown NB cells and control cells; (**E, F**) distributions of PDHA1 knockdown NB cells and control cells by cell cycle through flow cytometry. (p < 0.05 *; p < 0.01 **; p < 0.001 ***)

To determine the functional role of PDHA1 in NB cells, the siRNA targeting PDHA1 was transfected into SH-SY5Y and SK-N-AS cells. qRT-PCR and Western blot results on the 2nd, 7th and 12th day after transfection confirmed that si-PDHA1#1 and si-PDHA1#2 successfully downregulated PDHA1 in NB cells (Fig. 9A, Figure S3B and Figure S4). PDHA1 knockdown significantly reduced the proliferation and invasiveness of NB cells (Figure S5, Fig. 9B, C). Moreover, PDHA1 knockdown significantly attenuated the tube formation ability of HLECs incubated with the media collected from the NB cells (Fig. 9D). Furthermore, a flow cytometry assay suggested that the knockdown of PDHA1 blocked the NB cells in the G1 phase (Fig. 9E, F). Other than PDHA1, MTF1 and GLS were found negatively associated with poor prognosis in one dataset, while PDHB was positively associated with poor prognosis in one dataset (Fig. 1). MTF1 and PDHB knockdown significantly enhanced the cell proliferation of NB cells (Figure S6A, B, E, F), while GLS knockdown significantly attenuated the cell proliferation of NB cells (Figure S6C, D).

## Discussion

NB is among the most prevalent and malignant cancers in adolescents and children. It seriously affects both the mental and physical health of adolescents, which obviously has implications for their societal development [28]. Several new treatments have been developed for high-risk patients, which have improved NB prognosis; however, the survival rates for high-risk patients remain poor [3]. Therefore, further effective treatment strategies are urgently required to improve patient survival. Cuproptosis, which is a novel mode of apoptosis, has been recognized as being involved in disease progression and tumorigenesis in many cancers and can serve as a therapeutic target [29–31]. Nevertheless, the function of cuproptosis in NB has not yet been characterized. Therefore, this study strives to explore the mechanism of cuproptosis in the onset and progression of NB.

First, the individual effects of 10 key CUGs on the survival of NB patients were elaborated, and the results showed that PDHA1 associated significantly with poor prognosis in both two datasets. And PDHB were also positively associated with poor prognosis in NB patients in one dataset, while MTF1 and GLS were negatively associated with poor prognosis in one dataset. Other genes had no significant effect on survival. These results, thus, demonstrated that cuproptosis might influence NB progression. Subsequently, the NB patients were clustered and stratified into two subgroups according to the mRNA expression of the 10 key CUGs. Subgroup analysis revealed that the two subgroups exhibited different survival expectations, clinical features, and molecular pathways. Also, subgroup 1, which had a more negative survival outcome, was enriched significantly in tumor-associated signaling pathways, like the DNA replication pathway. Moreover, PDHA1 expression was upregulated in subgroup 1, as supported by previous findings.

Subsequently, to explore the molecular characteristics of the cuproptosis subgroup, an inter-group differential gene expression analysis was performed, which identified 53 NB prognosis-related DEGs. This was followed by the stratification of NB patients on the basis of prognostic DEGs. This resulted in stratified positive and negative signatures by the Boruta algorithm, named signature A and signature B, respectively. Thereafter, the cuproptosis scores of every patient were calculated, and they were classified into high- and low-score groups according to the median cuproptosis score value. The two groups exhibited different clinical features and survival curves; for instance, the low-score group was linked to poorer survival outcomes. UniCox and multiCox analyses further confirmed that cuproptosis could serve as an independent prognostic factor in NB patients and predict survival outcomes. The study also explored the association between the TIME and cuproptosis scores of both groups and concluded that they were weakly correlated. This suggests that cuproptosis may affect tumorigenesis and disease progression in NB without modulating the TIME. However, IF assays indicated that the tumor stages were positively associated with PDHA1 expression and negatively associated with CD56 expression in the NB tissues. CD56 is an important marker of NK cells [32]. Recent studies have revealed that NK cell activation suppresses the progression of NB [33]. Thus, it was hypothesized that high PDHA1 expression in NB cells might maintain the continuous survival of tumors via the mechanism of suppressing NK cell infiltration. However, further investigation is needed to confirm this hypothesis.

GSEA enrichment analysis revealed that cuproptosis is involved in the cell cycle and cell proliferation-related pathways in NB. Altogether, the results of our study found that PDHA1 is the most important CUG involved in NB. PDHA1, a critical component of the pyruvate dehydrogenase (PDH) complex (PDC), is critical in glucose metabolism because it participates in the tricarboxylic acid cycle and oxidative phosphorylation in mitochondria [34]. Huang T et al. showed that PDHA1 overexpression was linked to poor prognosis in breast cancer and that the expression of PDHA1 was closely related to the infiltrations of macrophage M0 and M1 cells, CD4 + memory T cells, and mast cells in breast cancer [35]. Another study showed that low expression of PDHA1 indicated poor prognosis in gastric cancer [36]. However, the role of PDHA1 in NB has not been sufficiently explored. Therefore, the role of PDHA1 in pan-cancer was determined, and its association with known oncogenes and key checkpoints in cell cycle- and cell proliferation-related pathways in NB was also explored. The significant positive correlation of these key nodes with PDHA1 further verified that PDHA1 was involved in cell cycle- and cell proliferation-related pathways and was also significantly involved in the progression and pathogenesis of NB. These results were further validated by in vitro experiments, which showed that PDHA1 could promote invasiveness, proliferation, and lymphatic metastasis of NB cells via the cell cycle pathway.

## Conclusion

In this study, a systematic approach was employed to analyze the impact of cuproptosis on NB and provided a clear explanation of the broad regulatory mechanisms of cuproptosis in NB TIME, clinicopathological characteristics, and prognosis. In addition, the results revealed the oncogenic role of PDHA1 in NB cells as well as the correlation between PDHA1 expression and tumor stages and NK cell infiltration in NB. These results suggest that an integrated assessment of cuproptosis scores for every NB patient is of significant clinical importance and can be applied to develop personalized immunotherapy techniques for these patients.

### Electronic supplementary material

Below is the link to the electronic supplementary material.

Supplementary Material 1

Supplementary Material 2

Supplementary Material 3

## Data Availability

Normalized data for gene expression (in Fragments/Kilobase of sequence/Million mapped reads [FPKM] format) and the comparable overall survival (OS) and clinicopathological data of the patients were collected from the GSE49710 dataset in GEO (n = 498, https://www.ncbi.nlm.nih.gov/geo/) and the GDC TARGET-NB datasets in UCSC Xena (n = 150, https://xena.ucsc.edu/). The original contributions presented in the study are included in the article/supplementary material. Additional inquiries can be directed to the corresponding authors.
