# Supplementary material for: Comprehensive exploration of the involvement of cuproptosis in tumorigenesis and progression of neuroblastoma
Source: BMC Genomics. 2023 Nov 27;24:715. doi: 10.1186/s12864-023-09699-2 (PMC10680286; doi:10.1186/s12864-023-09699-2)
Supplement: Supplementary file 1 — Supplementary Material 1 [file 12864_2023_9699_MOESM1_ESM.docx]

**Figure S1**

**Gene expression level of 10 CUGs in Cluster 1 and Cluster 2**


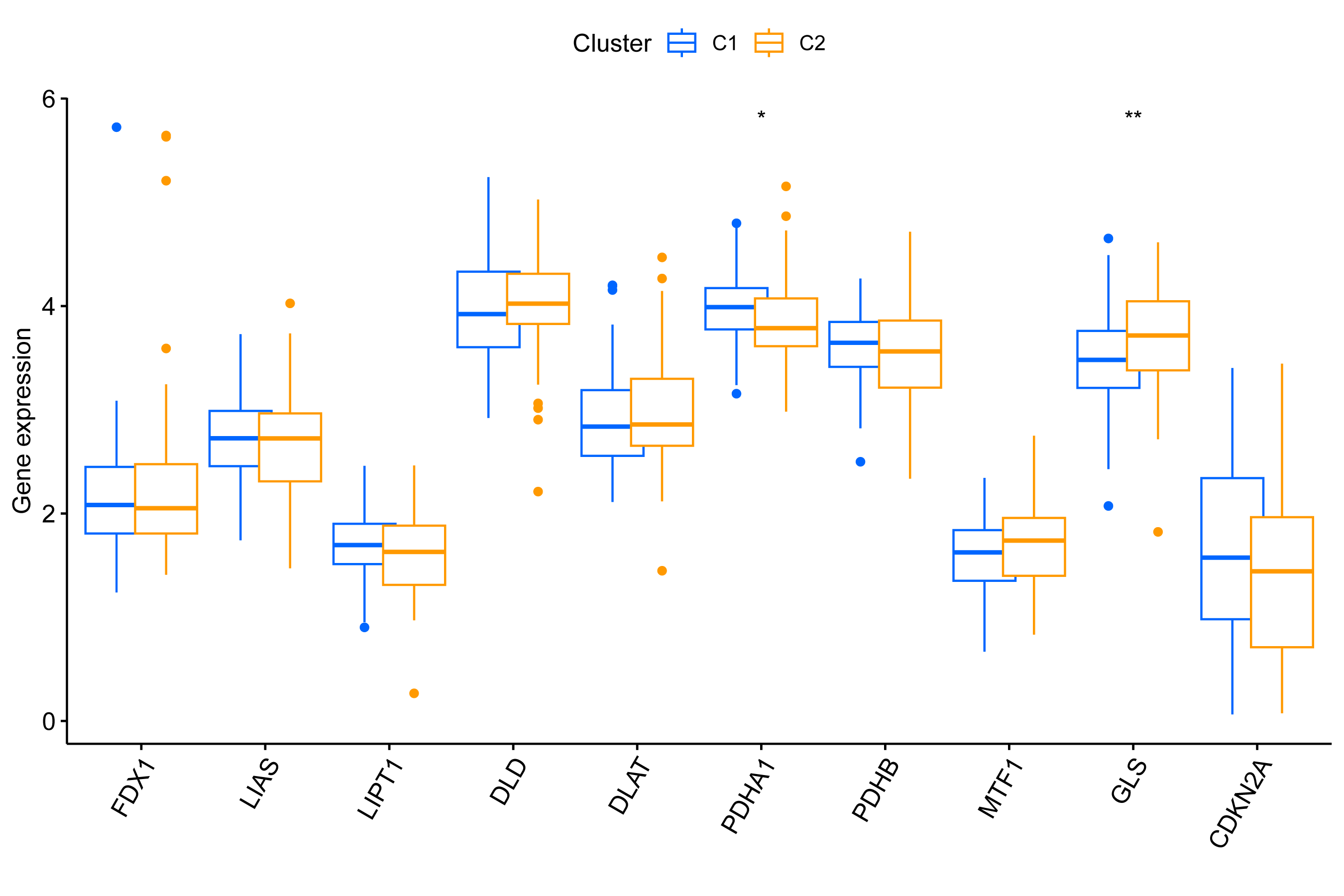


The expression of PDHA1 and GLS was higher in Cluster 2 than in Cluster 1. (p < 0.05 *; p < 0.01 **; p < 0.001 ***)

**Figure S2**

**Heatmap of three gene clusters**

**
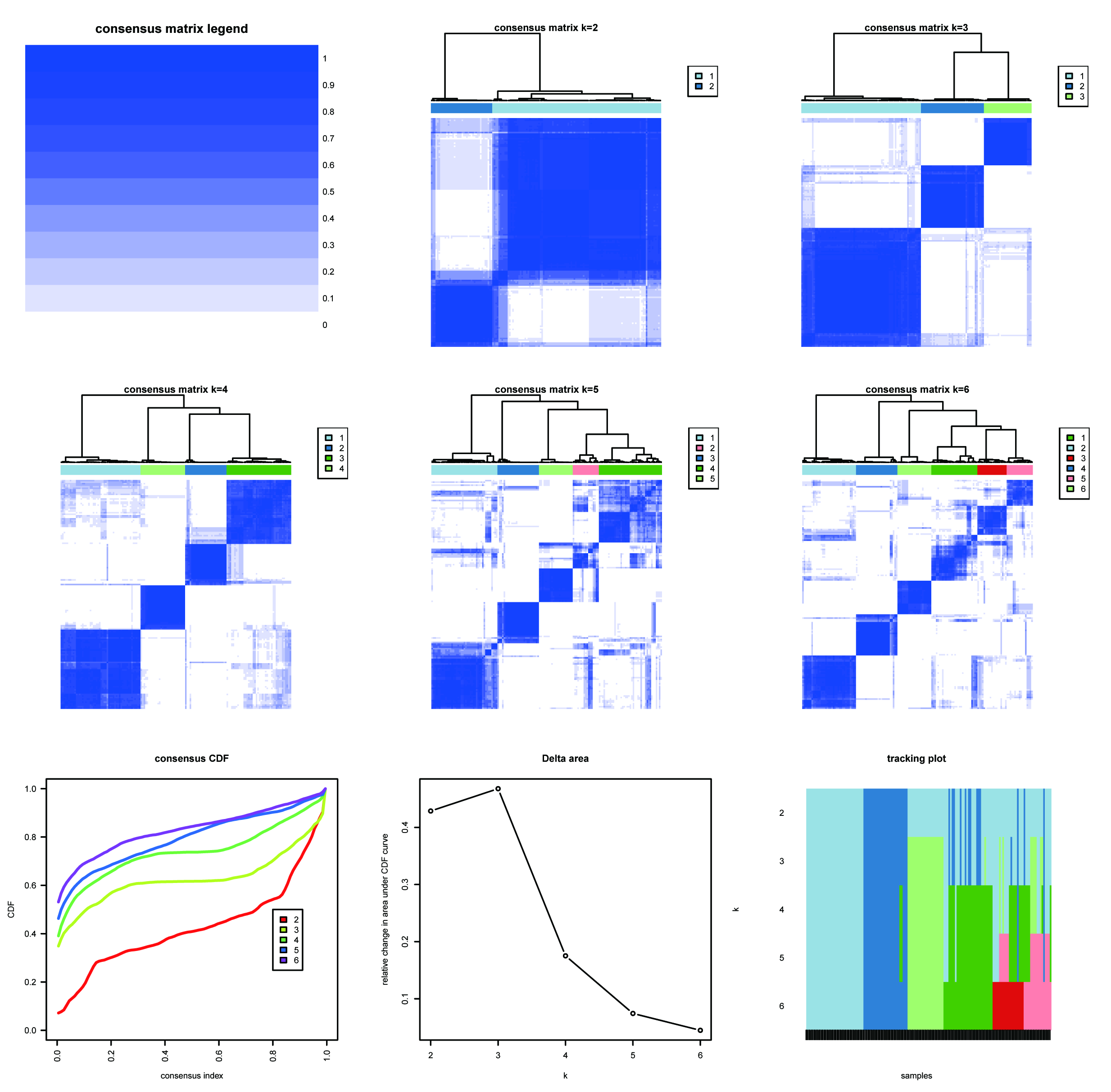
**

Consensus matrix heatmap displaying the three gene clusters as per their prognostic differentially expressed genes (DEGs)

**Figure S3**

**Relative expression of PDHA1 mRNA in NB tissues or cells by qRT-PCR**

**
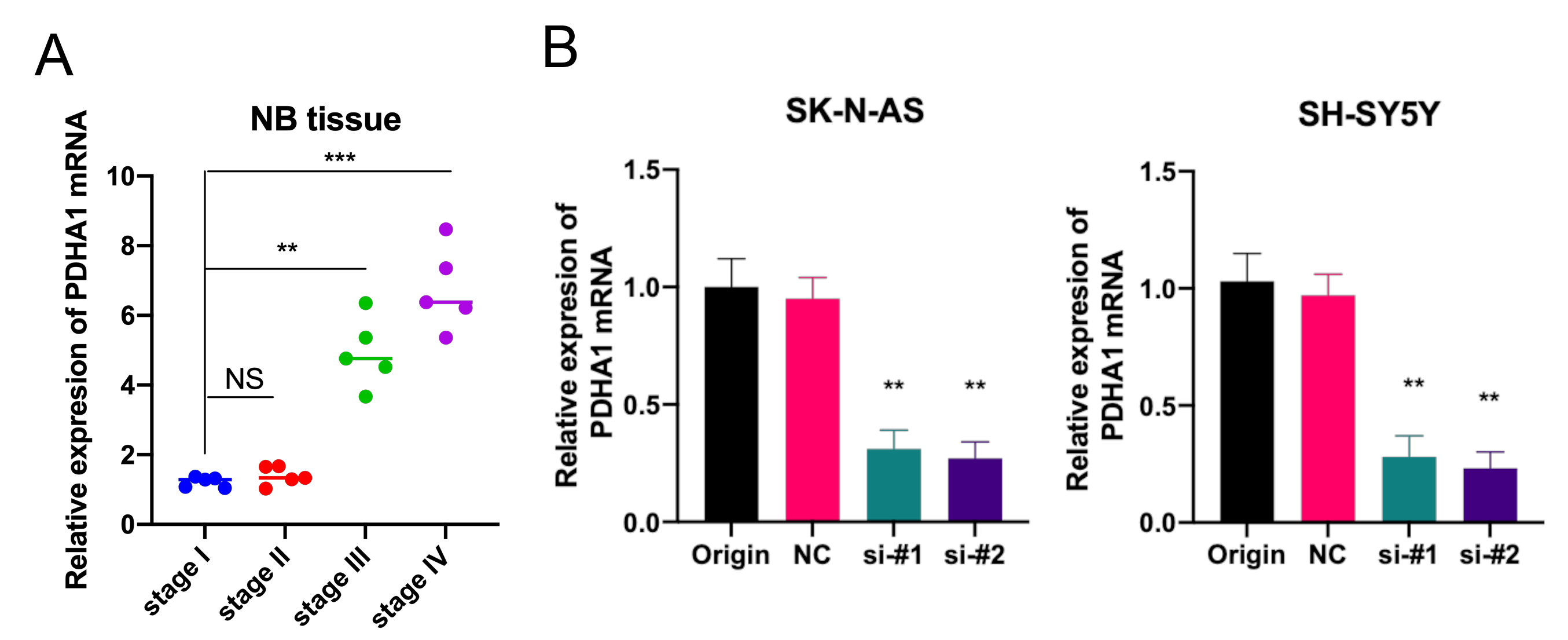
**

1. The mRNA level of PDHA1 in NB tissues was found positively correlated with the tumor stages through qRT-PCR. (p < 0.05 *; p < 0.01 **; p < 0.001 ***)
2. The mRNA level of PDHA1 in NB cells was significantly knocked down by si-PDHA1#1&#2. (p < 0.05 *; p < 0.01 **; p < 0.001 ***)

**Figure S4**

**The level of PDHA1 in NB cells on 7^th^ and 12^th^ day after si-RNA transfection by WB and qRT-PCR**

**
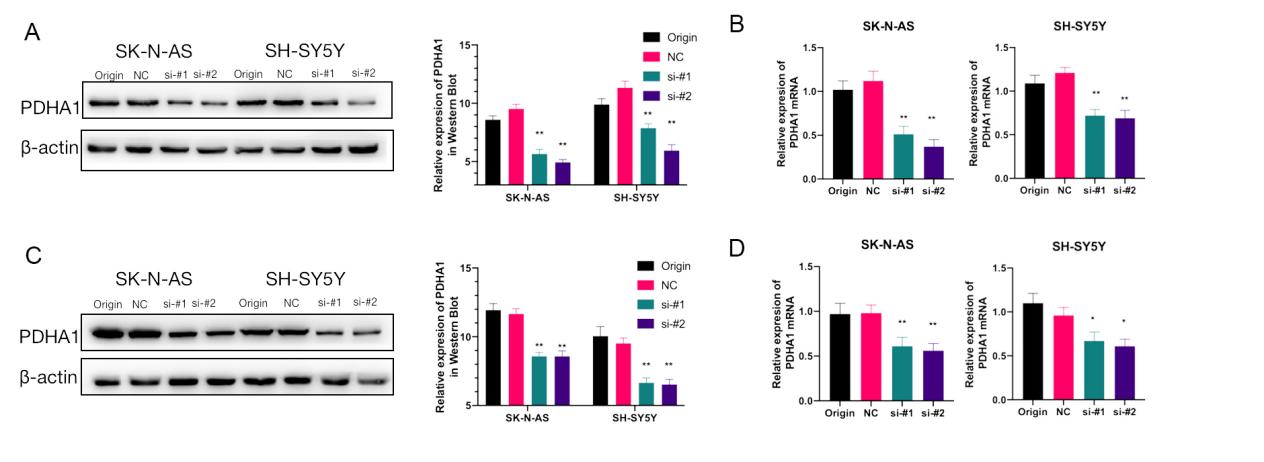
**

(A, C)The protein level of PDHA1 was significantly down-regulated in NB cells on 7^th^ and 12^th^ day after si-RNA transfection. (p < 0.05 *; p < 0.01 **; p < 0.001 ***)

(B, D)The mRNA level of PDHA1 was significantly down-regulated in NB cells on 7^th^ and 12^th^ day after si-RNA transfection. (p < 0.05 *; p < 0.01 **; p < 0.001 ***)

**Figure S5**

**Proliferation rate of NB cells after si-RNA transfection by CCK-8 assays**


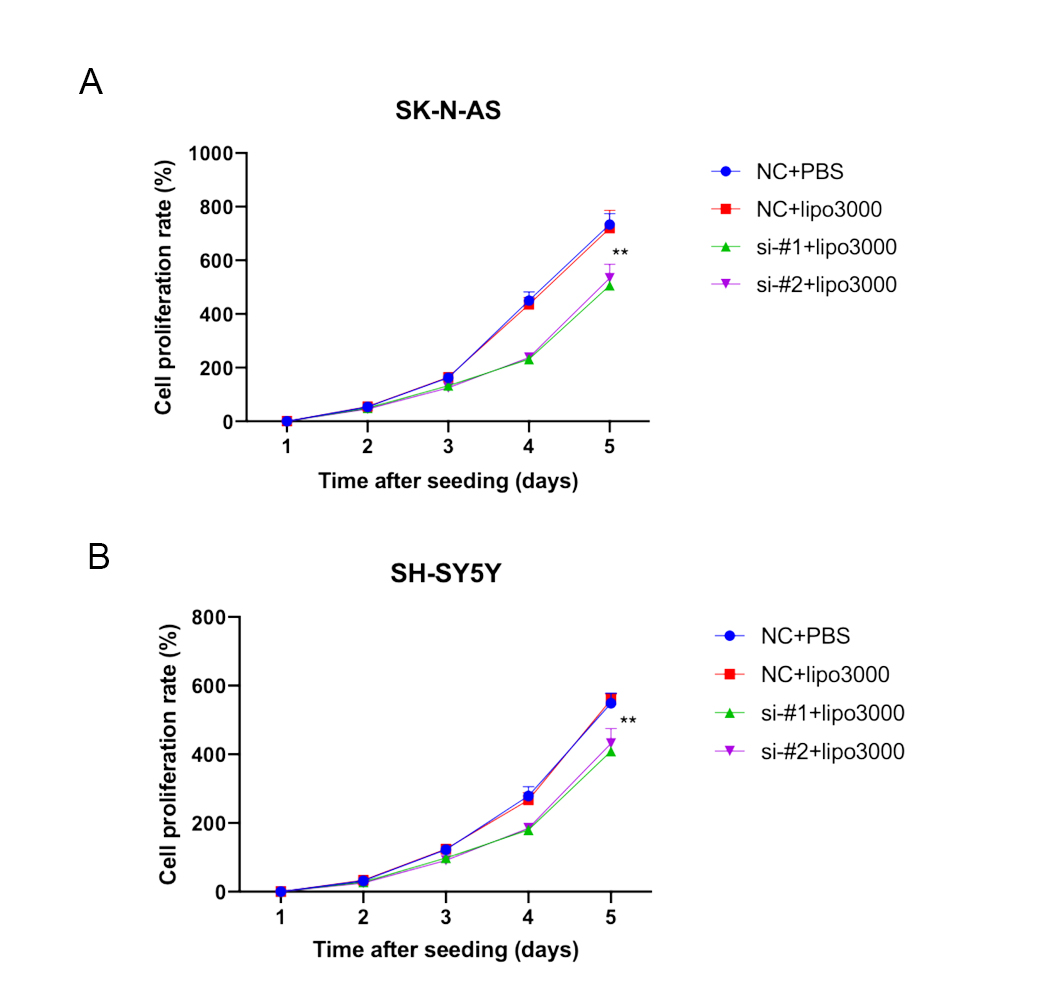


(A) (B) PDHA1 knockdown significantly reduced the proliferation of NB cells. Toxicity of lipofectamine 3000 did not significantly affect the proliferation of NB cells. (p < 0.05 *; p < 0.01 **; p < 0.001 ***)

**Figure S6**

**Targeting MTF1, GLS and PDHB influences the cell proliferation rate of NB.**

**
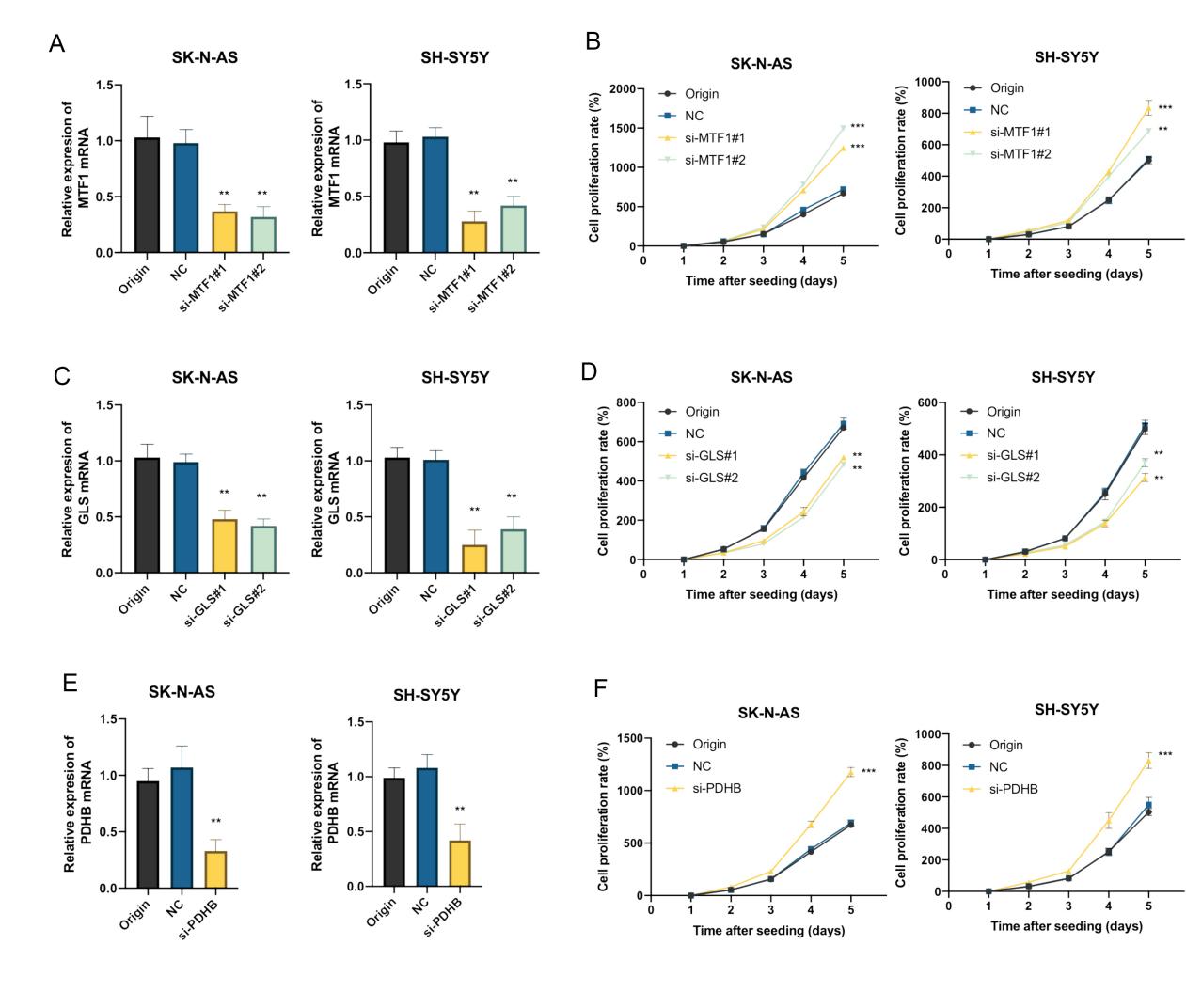
**

(A, B) The mRNA level of MTF1 was knocked down by si-MTF1 and significantly enhanced the cell proliferation rate of NB cells by CCK-8 assays. (p < 0.05 *; p < 0.01 **; p < 0.001 ***)

(C, D) The mRNA level of GLS was knocked down by si-GLS and significantly attenuated the cell proliferation rate of NB cells by CCK-8 assays. (p < 0.05 *; p < 0.01 **; p < 0.001 ***)

(E, F) The mRNA level of PDHB was knocked down by si-PDHB and significantly enhanced the cell proliferation rate in NB cells by CCK-8 assays. (p < 0.05 *; p < 0.01 **; p < 0.001 ***)
