# Supplementary material for: Comprehensive exploration of the involvement of cuproptosis in tumorigenesis and progression of neuroblastoma
Source: BMC Genomics. 2023 Nov 27;24:715. doi: 10.1186/s12864-023-09699-2 (PMC10680286; doi:10.1186/s12864-023-09699-2)
Supplement: Supplementary file 3 — Supplementary Material 3 [file 12864_2023_9699_MOESM3_ESM.docx]

**Supplemetary table 2**

**Sequences of si-PDHA1, si-MTF1, si-GLS and si-PDHB**

|  | Forward | Reverse |
| --- | --- | --- |
| si-PDHA1#1 | 5’-GAAUGGAGUUGAAAGCAGATT-3’ | 5’-UCUGCUUUCAACUCCAUUCTT-3’ |
| si-PDHA1#2 | 5’-GAGGAUCGAUGCACAUGUATT-3’ | 5’-UACAUGUGCAUCGAUCCUCTT-3’ |
| si-MTF1#1 | 5’-GGAAGAUCCUCAACAGACATT-3’ | 5’-UAUGUGUCCUGAAUUCCAGTT-3’ |
| si-MTF1#2 | 5’-GAAAGGUCAUGAUAACAAATT-3’ | 5’-UUAUGUGAUCGAACUCCAGTT-3’ |
| si-GLS#1 | 5’-GAUGGACAGAGGCAUUCUATT-3’ | 5’-UUAGAUGUGUCUUAACUCGTT-3’ |
| si-GLS#2 | 5’-CCCAGGUUGAAAGAGUGUATT-3’ | 5’-UGAUAUCGAUCGACCGCAGTT-3’ |
| si-PDHB | 5’-GGAAUUCAGAGGAUGCUAAAGTT-3’ | 5’-UUAGCAUCCUCUGAAUUCCAGTT-3’ |
| NC | 5’-GUGGAUAUUGUUGCCAUCATT-3’ | 5’-UGAUGGCAACAAUAUCCACTT-3’ |
